# Effect of tumor-associated macrophages on the pyroptosis of breast cancer tumor cells

**DOI:** 10.1186/s12964-023-01208-y

**Published:** 2023-08-04

**Authors:** XuLing Ji, Xiaoxia Huang, Chao Li, Ningning Guan, Tingting Pan, Jing Dong, Lin Li

**Affiliations:** 1 College of Animal Science and Veterinary Medicine, Shenyang Agricultural University, Shenyang, 110866 China; 2 Key Laboratory of Livestock Infectious Diseases, Ministry of Education, Shenyang Agricultural University, Shenyang, 110866 China

**Keywords:** Tumor-associated macrophages, Pyroptosis, Breast cancer, Cytokines, Tumor immune microenvironment

## Abstract

Macrophages are immune cells with high plasticity that are widely distributed in all tissues and organs of the body. Under the influence of the immune microenvironment of breast tumors, macrophages differentiate into various germline lineages. They exert pro-tumor or tumor-suppressive effects by secreting various cytokines. Pyroptosis is mediated by Gasdermin family proteins, which form holes in cell membranes and cause a violent inflammatory response and cell death. This is an important way for the body to fight off infections. Tumor cell pyroptosis can activate anti-tumor immunity and inhibit tumor growth. At the same time, it releases inflammatory mediators and recruits tumor-associated macrophages (TAMs) for accumulation. Macrophages act as “mediators” of cytokine interactions and indirectly influence the pyroptosis pathway. This paper describes the mechanism of action on the part of TAM in affecting the pyroptosis process of breast tumor cells, as well as its key role in the tumor microenvironment. Additionally, it provides the basis for in-depth research on how to use immune cells to affect breast tumors and guide anti-tumor trends, with important implications for the prevention and treatment of breast tumors.

Video Abstract

**Supplementary Information:**

The online version contains supplementary material available at 10.1186/s12964-023-01208-y.

## Introducton

Breast cancer is the most common type of cancer among female cancer patients worldwide. Over the past two decades, breast cancer trends have consistently shown an increase. According to the statistics of the percentage change of total global mortality caused by non-communicable diseases, the mortality rate of breast cancer increased from 14.9% to 27.2% between 2005 and 2015, with an increase of 534,000 deaths [1]. In 2016, a study of breast cancer prevalence and mortality in 195 countries was conducted, showing that Latin America and the Caribbean super-region had the highest growth rates and that African countries had lower growth rates, but the outlook is not promising under the influence of an increasing aging population [2]. In the most recent study, breast cancer, although still the most common cancer among women, had an increased proportion of survivors due to advances in diagnostic methods and more comprehensive implementation of screening programs [3]. Despite the development and diffusion of breast imaging technology, which has improved the early detection rate of breast cancer patients, the treatment and prognosis of breast cancer remains a major challenge. The physical damage and psychological shadow of this malignant disease is indelible for patients. Prolonged drug therapy and chemotherapy can produce cardiotoxicity, fertility damage, osteoporosis and other complications [4]. Pain, fatigue, sexual dysfunction, and lymphedema exacerbate the patient's suffering and reduce quality of life. Body image destruction, premature menopause, feelings of isolation and fear of disease recurrence increase patients' odds of developing anxiety disorders [5]. These treatment complications have a significant impact on the patient's life.

Tumorigenesis is associated with mutations, amplifications, or deletions of critical genes that regulate cell growth and differentiation, including proto-oncogenes, oncogenes, and genes that affect DNA repair [6]. In addition to this, tumor growth and proliferation are influenced by the mechanism of cancer cell death. Initially, cells were found to exhibit characteristics of apoptosis and necrosis, and as the mode of death of cancer cells became better understood, multiple modes of death were also observed that undergo different processes from the former morphological and molecular mechanisms. The genetically regulated, orderly way of death that helps to maintain the stability of the body is called regulated cell death (RCD), such as apoptosis, necroptosis, pyroptosis, ferroptosis, autophagy, NETosis, parthanatos, etc. [7]. These modes of death can affect tumor inflammation and immunity through the release of danger signals, cytokines, metabolites, and other biomolecules [8]. The relationship between pyroptosis and cancer and immunity has received much attention as GSDM proteins have been intensively studied. A growing body of research evidence suggests that pyroptosis has an important role in tumors. Tumor cell pyroptosis is closely associated with tumor growth and invasive metastasis and is also able to regulate the tumor microenvironment [9]. Tumor-associated macrophages (TAM), a primary component of tumor inflammatory infiltration, are cytotoxic to tumor cells on the one hand and interact with pyroptosis signaling pathways on the other. Therefore, targeted regulation of TAM metabolism for anticancer therapy is particularly important to understand the role of TAM on the tumor microenvironment and the role it plays in the pyroptosis pathway. It has been shown that related genes act as agonists of macrophage pro-inflammatory activation, modulating macrophage function and promoting or inhibiting disease progression [10]. In the next section, we describe the pathways and mechanisms of action of TAM on breast cancer cell pyroptosis, and their role in cancer development and immune regulation.And we discuss that these pathways offer new directions for finding effective potential therapeutic targets, improving cancer treatments, and reducing drug resistance and side effects.

### TAM source and function

Macrophages are present in various tissues of the body, and they are an important component of the immune barrier. Fetal-derived macrophages are generated from primitive hematopoietic progenitor cells during the yolk sac developmental stage [11]. The analysis of yolk sac macrophage phenotypes and the differentiation process shows that macrophage growth and development are divided into three waves. The initial maternal macrophage differentiates and proliferates to a certain stage; appears as mononuclear macrophage precursors; and, finally, produces red myeloid cell precursors [12]. Mononuclear cells that enter the bloodstream develop and mature in different tissues. This is a form of macrophage recruitment that contributes to the homeostasis of the body and the fight against disease [13]. The classification of the macrophage system is very complicated, and it is broadly divided into two types according to metabolic function. M1 macrophages have anti-inflammatory and anti-tumor effects due to the increased secretion of inflammatory cytokines caused by microbial infections. M2 macrophages, which have collagen fiber repair functions, produce immunosuppressive cytokines; participate in angiogenesis, tissue repair, and immunosuppression; and promote tumor production [14]. In tumors, macrophages are involved in tumorigenesis, development, and metastasis and are also known as tumor-associated macrophages (TAMs). During the development of breast cancer, TAMs can affect angiogenesis, invasion, metastasis, and tumor immunosuppression [15].

### Breast cancer

Women under the age of 40 have a progressively higher prevalence of breast cancer and exhibit a higher risk of mortality and recurrence [16]. The Swedish breast cancer risk prediction model also aims to detect breast cancer early and provide prevention and treatment strategies [17]. The treatment options for breast cancer patients at different ages and stages are different.The main methods are chemotherapy, radiation therapy, endocrine therapy, molecular targeted therapy and other supportive treatments.

Breast cancer are insidious in the early stage of development and often do not have obvious clinical symptoms. At this time, the lumps are already visible in the breast area. Malignant tumors can be seen to rupture in advanced stages, with blood-like or pus-like fluid flowing from the surface. A routine examination is performed by measuring body temperature, heart rate, breath, and palpating the size, location, softness and pain of the mass to determine the abnormalities. Further examination is required by hematological tests, biochemical index tests, X-ray examinations, ultrasound examinations, and pathological biopsy methods [18]. The newly established DW-MRI diagnostic modality can enhance tissue resolution by dynamic contrast and has higher sensitivity than conventional imaging techniques. Advances in breast cancer screening have been driven by the development of artificial intelligence in the field of cancer, combining data analysis and x-ray technology, which has provided additional opportunities for early diagnosis and treatment of breast cancer [19].

Patients with breast cancer detected at an early stage have increased chances of being treatable. Traditional endocrine therapy is considered to be one of the most commonly effective treatments for breast cancer patients. Tamoxifen, for example, an estrogenic drug, may have a preventive and therapeutic effect on breast cancer by inhibiting the proliferation of tumor cells. Most patients develop acquired resistance to these drugs over time, and some patients also experience adverse effects such as thromboembolism, uterine fibroids, and cardiac arrhythmias [20]. Chemotherapy and radiotherapy have been shown to be effective in cancer treatment and prognosis improvement, but the damage caused to liver and kidney function and other normal tissues is irreversible. In low-risk breast cancer, chemotherapy reduces local recurrence but does not reduce mortality [21]. Target-specific drug therapy is often more efficient and has fewer side effects than conventional therapies such as hormone therapy and chemotherapy. Molecularly targeted therapies often target molecular pathways or specific genes, such as compounds targeting PI3K, CDK4/6 inhibitors [22].

Immunotherapy for tumors has made great progress, with extensive clinical research and applications. In particular, research on immunotherapy response factors and combination therapy strategies has attracted attention. Various immune cells play a major role in the pathogenesis of breast cancer, among which TAMs can perform a variety of functions. These TAMs can regulate the growth factors, chemokines, and pathogenic factors in the immune microenvironment, as well as playing an important role in immunotherapy [23]. In addition, it is necessary to study the interactions of focal death-related molecules in the immune microenvironment in breast cancer. On the one hand, it helps to in creating a prognosis for breast cancer, and on the other hand, it provides new and more precise targets for breast cancer treatment [24].

### Pyroptosis

Pyroptosis is a type of inflammatory cell death that is distinct from apoptosis and has the morphological characteristic of cell swelling. Various factors cause the cleavage of the GSDM gene family proteins, releasing the N-terminal structural domain and forming transmembrane pores in the cell membrane. Cells become swollen after perforation and release cytokines to the outer membrane. This process causes a severe inflammatory response, with immune effects in the extracellular fluid [25]. The Gasdermin family proteins include Gasdermin A, Gasdermin B, Gasdermin C, Gasdermin D, Gasdermin E, and DFNB59. These are differentially expressed in different species and tissues, and their pore-forming properties play a key role in the pyroptosis process and inflammation [26]. In antitumor-related studies, three proteins, GSDMA, GSDMD, and GSDME, have been most frequently studied [27]. Bacterial proteins can cleave GSDMA and trigger pyroptosis, and GSDMD can be cleaved by caspase-1, caspase-4, caspase-5, caspase-8, and caspase-11, releasing N-terminal fragments that cause pyroptosis via classical or non-classical pathways [28]. Caspase-3, caspase-8, and granzyme all cleave GSDME, causing cells to swell and rupture, releasing cytokines [29].

The classical pyroptosis pathway is mediated by inflammatory vesicles, which are made active at the nitrogen end by cleavage of GSDMD, generating pores in the cell membrane where cells release inflammatory factors such as IL-1β and IL-18 [30]. When stimulated by different pathogens or infections, the corresponding intracellular receptors are activated to assemble different inflammatory vesicles, activate Caspase-1 and cause secondary shearing of GSDMD proteins. At this point the corresponding pro-inflammatory cytokines are converted from precursors to a mature mode and released from the pores generated by the GSDMD protein, causing an inflammatory response. Among them, NLRP3 inflammatory vesicles are essentially species cytoplasmic multimeric protein complexes, which are key factors in the onset of pyroptosis and promote the activation of caspase-1. Inflammatory vesicle complexes are formed mainly by certain components of the nucleotide-binding structural domain, proteins containing leucine repeat sequences (NLR) and AIM2-like receptors (ALR), pathogen-associated molecular patterns (PAMP) or damage-associated molecular patterns (DAMP). In which PAMP and DAMP act as the initiator receptor part of the inflammatory vesicle complex linked to the bridging protein ASC, and the resulting complex is then linked to the Caspase-1 protein [31]. Caspase-1 is activated and cleaves the GSDMD protein, producing a C-terminal fragment (GSDMD-CT) and an active N-terminal cleavage product (GSDMD-NT). GSDMD-CT is thought to be in an inhibited state that is difficult to activate, and GSDMD-NT binds phosphatidylinositol phosphate and phosphatidylserine and cardiolipin, which accumulate on the cell membrane and are pores that form on the surface of the cell membrane, the size of which can be observed microscopically [32]. Meanwhile, caspase-1 induces the precursors of IL-1β and IL-18 to become mature IL-1β and IL-18, which are secreted extracellularly through the pores formed by perforin after cell membrane perforation, inducing inflammatory responses to occur and activating the pyroptosis of other surrounding cells [33].

Non-classical cell pyroptosis pathways are those directed by activation of caspase-4, 5, and 11, which are mediated by caspase-4/5 in humans and by caspase-11 in mice. Caspase-4, 5, and 11, like caspase-1, can be induced by LPS to release active GSDMD-NT by cleavage with GSDMD as a substrate [34]. LPS can promote the upregulation of various intracellular inflammatory cytokines that also play a key role in the development and regulation of inflammation [35]. Intracellular LPS-induced pyroptosis in the form of GSDMD cleavage with release of IL-1β and IL-18 often plays a protective role during bacterial infection, but excessive pyroptosis can also negatively affect disease or organismal recovery [34]. It was found that the activation of proteins such as Caspase-11 could be delayed by regulating the pre-initiation of LPS, affecting the rate and extent of pyroptosis [36].

Pyroptosis is involved in the development of many diseases, including asthma, diabetes, autoimmune diseases, ischemic stroke, Parkinson’s disease, and atherosclerosis.Pyroptosis has a complex mechanism of action in cancer. On the one hand, it can be involved in the release and utilization of various cytokines under the joint action of caspase and Gasdermin family proteins. These cytokines promote tumor angiogenesis by exacerbating hypoxic and inflammatory responses through different pathways. Pyropsis causes the activation and aggregation of immune cells and aggravates tumor development. On the other hand, pyroptosis can induce tumor cell death, enhance immunity, and resist the invasive effect of tumor cells.

### Cell death pathways and breast cancer

During the development of the organism, cells are constantly proliferating and cell death is occurring simultaneously in different ways, which may result in different ways. In addition to cell death caused by pathological factors such as pathogenic infections, metabolic disorders, and cellular stress, cell death is programmed under normal physiological conditions controlled by different signaling pathways that regulate unwanted cell death in the body and are removed. Programmed cell death is an essential life process in the internal environment of the organism, including fetal development and growth and regulation of tissue homeostasis [37]. Programmed cell death mainly includes apoptosis, pyroptosis, autophagy, necroptosis and ferroptosis, etc. The pathways of these death modalities are quite flexible and their molecular regulation also exhibits a considerable degree of plasticity [38]. More than a decade ago, the molecular mechanisms and effects of apoptosis in tumor cells have been studied in depth. Apoptosis is regulated by cysteine protease signaling in response to stimulation by exogenous or endogenous pathways, with morphological features such as apoptotic vesicle formation, chromatin crinkling, and DNA fragmentation in chromosomes [39]. In the apoptotic signaling pathway of breast cancer cells, tumor necrosis factor (TNF) mediates the extrinsic pathway for intra- and extracellular signaling. In the mitochondria-dependent apoptotic signaling pathway, apoptotic factors are regulated by a combination of pro- and anti-apoptotic proteins and are released into the cytoplasm to cause apoptosis. Among them, the anti-apoptotic protein Bcl-2 family proteins show overexpression in breast cancer cells, inhibit apoptosis, and promote tumor growth [40]. Cellular autophagy has a dual role in breast cancer, acting as a killer during tumor proliferation on the one hand, and providing the nutrient-deficient tumor cells with substances and energy for metabolism on the other. Among them, the PI3KC1-Akt-mTORC1 signaling pathway is one of the key pathways regulating autophagy and is often used for targeted therapy to inhibit tumor cell proliferation [41]. Necroptosis is mainly mediated by mixed-spectrum kinase structural domain-like protein (MLKL), receptor-interacting protein kinase 1 (RIPK1) and RIPK3, causing enhanced cell permeability and mitochondrial modifications [42]. In breast cancer, necroptosis affects cancer progression, accelerates tumor metastasis, is closely associated with malignant tumor markers, and promotes epithelial-mesenchymal transition (EMT) and angiogenesis [43]. The activation of iron death is associated with the absence of glutathione peroxidase 4 (GPX4) activity. It is characterized by phospholipid peroxidation reactions, metabolic abnormalities, and glutathione deficiency [44]. Because breast cancer cells are highly proliferative and metabolically active, they are more sensitive to iron death. Meanwhile, multiple oncoproteins and tumor suppressors promote this phenomenon and iron death proteins such as GPX4, ACSL4 and PTGS2 can be utilized in anti-tumor [45]. In breast cancer cells, the expression levels of NLRP3 and IL-1β are significantly higher compared to normal tissues, contributing to the occurrence of pyroptosis in the tumor microenvironment. Scorch death, as a form of inflammatory cell death, releases large amounts of inflammatory factors such as IL-1β and IL-18, which are released into the tumor microenvironment [46]. This suggests that drugs can achieve anti-tumor immune effects by targeting breast cancer pyroptosis-related factors.

### TAMs are involved in breast tumor cell pyroptosis

Gasdermin family proteins are induced to activate during cell pyroptosis. Cell membranes are damaged, forming membrane pores, which, in turn, cause cell swelling and death. Tumor-associated macrophages are involved in pyroptosis through the classical pathway induced by the caspase-1 cleavage of GSDMD and the pathway mediated by the caspase-8 cleavage of GSDME and the granzyme action of GSDMB. In this process, the regulation of inflammasomes, granzymes, and cytokines plays a key role.

In the caspase-1-dependent classical pyroptosis pathway, related molecular patterns (PAMPs and DAMPs) are recognized by pattern recognition receptors (PRRs) in response to stimuli induced by various factors. These PRRs include Toll-like receptors (TLRs) and Nod-like receptors (NLRs). The activation of the protein complex inflammatory vesicle NLRP3 by PRR causes GSDMD-induced cell pyroptosis [47]. At the corresponding signals, GSDMD is activated by caspases 1, 4, 5 and 11. GSDMD is cleaved at two sites, Asp276 and Asp275, to generate the products GSDMD-NT and GSDMD-CT. Among them, GSDMD-NT is concentrated on the cell membrane and binds to phosphatidylinositol phosphate, phosphatidylserine, cardiolipin, and other lipids on the cell membrane. Then, a membrane hole is formed, triggering pyroptosis [32].

The mechanism of activation of inflammatory vesicles is divided into two processes. In both processes, macrophages can regulate the onset of breast cancer cell pyroptosis, which is mediated by inflammatory vesicles through the release of relevant inflammatory factors. In the first process, microbial infection and pro-inflammatory cytokines, such as IL-α and IL-β, trigger the NF-κB activation pathway and promote the production of pro-IL-1βand pro-IL-18 [48]. NF-κB is activated by the TAM-secreting cytokine IL-β [49] and also by bacterial, viral, lipopolysaccharide, physical and chemical stimuli. NF-κB is activated to regulate a variety of genes and thus promote tumor growth, migration, angiogenesis, and pro-apoptotis or pyroptosis through various pathways [50]. The granulocyte macrophage colony-stimulating factor (GM-CSF) gene also plays an important role in the NF-κB signaling pathway, inducing the osteolytic metastasis of breast cancer cells [51]. In the second process, when cells are damaged, DAMPs released by various organelles, especially mitochondria, are recognized by TLRs on the cell surface, causing intracellular mitochondrial ROS release and K + efflux [52]. Various pathogenic factors can stimulate the release of pro-inflammatory mediator HMGB1 from TAMs [53]. In the tumor microenvironment, HMGB1 is a typical DAMP that is involved in the activation of inflammatory vesicles. In addition, it can improve the sensitivity of breast cancer tumor cells to drug and radiation therapy [54]. TAMs promote the release of ATP and act on P2X7 receptors on the cell surface, opening cell membrane cation channels, decreasing intracellular K + , and promoting inflammatory vesicle activation [55]. The oxidative stress-induced generation of reactive oxygen species also upregulates NLRP3, which mediates caspase-1-induced pyroptosis [56]. By establishing a mouse mammary tumor model, the TAM high-expression gene CD11b was isolated via FCAS screening and expression analysis. The reduction of NLRP3 in the S1PR1-KO CD11bhi TAMs group as compared with the WT group decreased IL-1β levels in tumor extracellular fluid more significantly. This suggests that S1PR1 signaling in TAMs also plays a role in the activation of inflammatory vesicles [57]. This evidence suggests that TAMs are more than sufficient to act on signaling molecules at various stages of inflammatory vesicle activation and promote the pyroptosis process.

In addition to classical pyroptosis, atypical pyroptosis pathways are also highly associated with TAMs. The inhibition of TGF-activated kinase 1 (TAK1) because of bacterial infection leads to the silencing of the mitogen-activated protein (MAPKs) pathway. This causes the caspase-8 cleavage of GSDMD and GSDME-induced pyroptosis, and its inflammatory vesicles enter the TAMs activated by TAK1, prompting pro-IL-1 to generate IL-1 [58]. TAK1-activated kinase can be activated by cytokines such as TNFa and IL-1. Then, TNFa receptor type 1-associated death domain protein (TRADD), an inhibitor of apoptosis (cIAP), and TNFa receptor-associated factor (TRAF) are recruited to form a linear ubiquitin chain. TAK1 is activated by binding to TAK1-binding protein 2 (TAB2) in the ubiquitin chain. This process activates the NF-KB pathway, and PIPK1 binds to caspase-8 and FADD to form a cytoplasmic protein complex, which mediates cellular pyroptosis [59]. Granulocyte colony-stimulating factor (GCSF) and granulocyte macrophage colony-stimulating factor (GM-CSF) can activate caspase-8 through the PI3K/Akt pathway. Activated caspase-8 drives TAM differentiation and cleaves RIP1 to regulate intranuclear NF-κB transcription [60]. MicroRNA-155-3p can be delivered by the secretome of M2 macrophages [61]. As a novel prognostic biomarker, MicroRNA-155-3p is an endogenous non-coding single-stranded RNA molecule closely related to tumors [62]. The comparison of primary breast cancer tissue with normal tissue suggests the high expression of MicroRNA-155-3p [63]. MicroRNA-155-5p has been shown by TargetScan and luciferase reporter gene assay to be a potential binding target for GSDME, which can affect pyroptosis through its own regulation [64]. GSDME expression increases the phagocytic capacity of TAMs and the cytotoxic T cell population and enhances anti-tumor immunity [65]. Thus, the signaling molecules released by TAMs could also be involved in promoting the pyroptosis of GSDME cleavage.

**Fig. 1:**
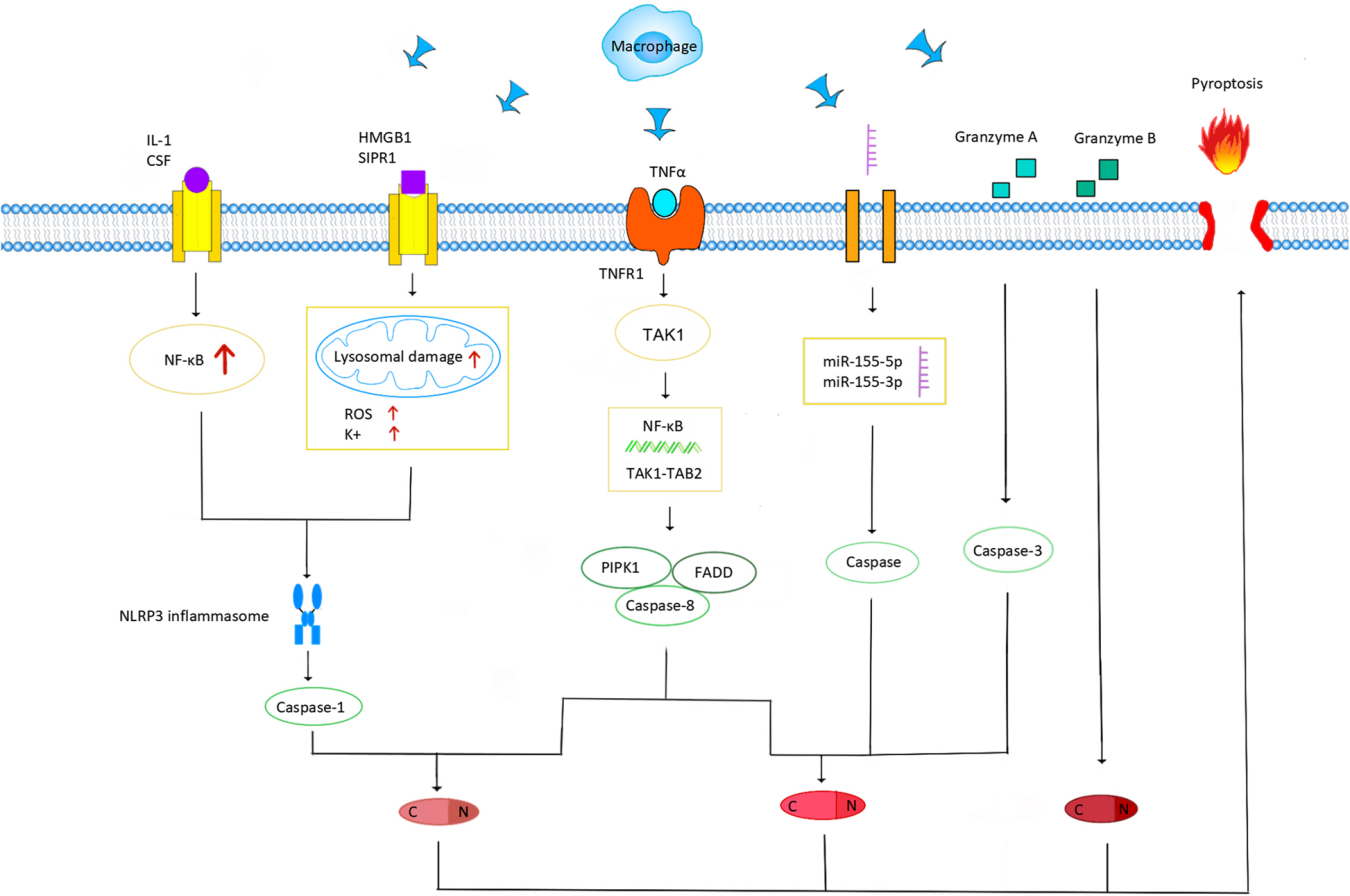
A schematic diagram of TAM involvement in the pyroptosis process of breast cancer cells: TAM is involved in pyroptosis mediated by Gasdermin family proteins through multiple pathways, including typical and atypical pathways

Granzyme B also activates caspase-3, which effectively cleaves GSDME and leads to pyroptosis. This process promotes the release of pro-inflammatory cytokines from TAM, triggering cytokine release syndrome (CRS), which, in turn, affects the pyroptosis process [66]. Granzyme B is present in a variety of cells, including T cells, NK cells, B cells, macrophages, dendritic cells, and basophils, and is a serine protease [67]. CD4- and CD8-positive TAMs express high levels of granzyme and perforin, which flow into target tumor cells with calcium ions [68]. Granzyme B can directly cleave GSDME and also indirectly cleave GSDME by activating caspase-3 to attack tumor cells and inhibit the invasiveness of breast cancer tumors [69]. In contrast, the cleavage of GSDMB by granzyme A is less effective in suppressing tumors. The high expression of GSDMB in breast tumors increases the rate of metastasis and decreases survival [70]. In particular, it plays a key role in the treatment and prognosis of human epidermal growth factor receptor 2-positive breast cancer [71] (Fig. 1).

### Effect of tumor microenvironment on TAM

Various signaling molecules are secreted or metabolized between cells in the breast tumor microenvironment. These cytokines regulate TAM polarization, recruitment, aggregation, tumor angiogenesis, and TAM phagocytosis. This, in turn, affects the involvement of TAMs in the pyroptosis process and produces pro- or anti-tumor effects.

Interferon gamma (IFN-γ), lipopolysaccharide (LPS), and GM-CSF are important factors that regulate TAM (M1) polarization with tumor-killing cytotoxicity. Lipopolysaccharide binds to TLR4 on the cell membrane surface and plays an inductive role, together with IFN-γ. In addition, nitric oxide (NO) and reactive oxygen species (ROS) production are two indicators of M1 polarization [72]. Cytokines associated with TAM (M2) polarization include CSF-1, transforming growth factor (TGF-β), IL-4, IL-10, and IL-13 [73]. Tumor-associated macrophages are affected by polarization, leading to various phenotypes that correlate strongly with the ability to hinder cancer cell growth and reproduction.

TAMs are regulated by chemokines during aggregation and invasion, and they are involved in the growth and metastasis of breast cancer cells [74]. The evaluation of serum growth factor levels in breast cancer patients revealed high levels of macrophage colony-stimulating factor (CSF) in early-stage patients. Such CSF can affect the release of inflammatory substances from macrophages. Colony-stimulating factor is a tyrosine kinase that shows the highest level of coordinated expression in assessing the expression of CSF-responsive genes in breast cancer cells. CSF1 receptor (CSFR) expression in TAMs is far superior to that in tumor cells [75]. It stimulates TAM polarization and can generate signals to recruit TAMs and dendritic cells, which play an important role in the mechanism of breast carcinogenesis [76]. CX3CL1, a transmembrane protein, is activated by fibroblast growth factor receptor 1 (FGFR) of breast tumor cells to bind to CX3CR1 on the surface of TAM membranes and mediates TAM aggregation. It can act on various immune cells, plays a complex role in various tumor microenvironments, promotes early breast cancer development, and is one of the markers of breast cancer risk and prognosis [77]. CC-chemokine ligand 2 (CCL2) is also capable of recruiting TAMs, and the cascade response triggered promotes breast cancer metastasis. CC-chemokine ligand 2 promotes tumor cell extravasation by recognizing the CCL2 receptor (CCR2) in TAM and secreting the ligand CCL3 [78].

Breast cancer development through the secretion of vascular endothelial growth factor (VEGF), transforming growth factor (TGF-β1), and other signaling molecules. These signaling molecules can stimulate abnormal blood vessel growth and play an important role in the infiltration and promotion of tumor cells [79]. Tumor-assisted macrophages and other immune cells kill tumor cells to release vasopressor and are also stimulated to secrete IL-1, VEGF, metalloproteinases, and other factors that promote angiogenesis in breast cancer tumors. This process is significantly upregulated by hypoxic stress in the tumor microenvironment [80]. Tissues alleviate hypoxia by regulating the mitochondrial production of large amounts of ROS [81]. The ROS accumulation caused by adriamycin (DOX) treatment in HER2-positive breast cancer tissues causes the phosphorylation of JNK and the caspase-3 cleavage of GSDME-induced pyroptosis [82]. This suggests that ROS are closely associated with both caspase-1 and caspase-3-induced pyroptosis.

**Fig. 2:**
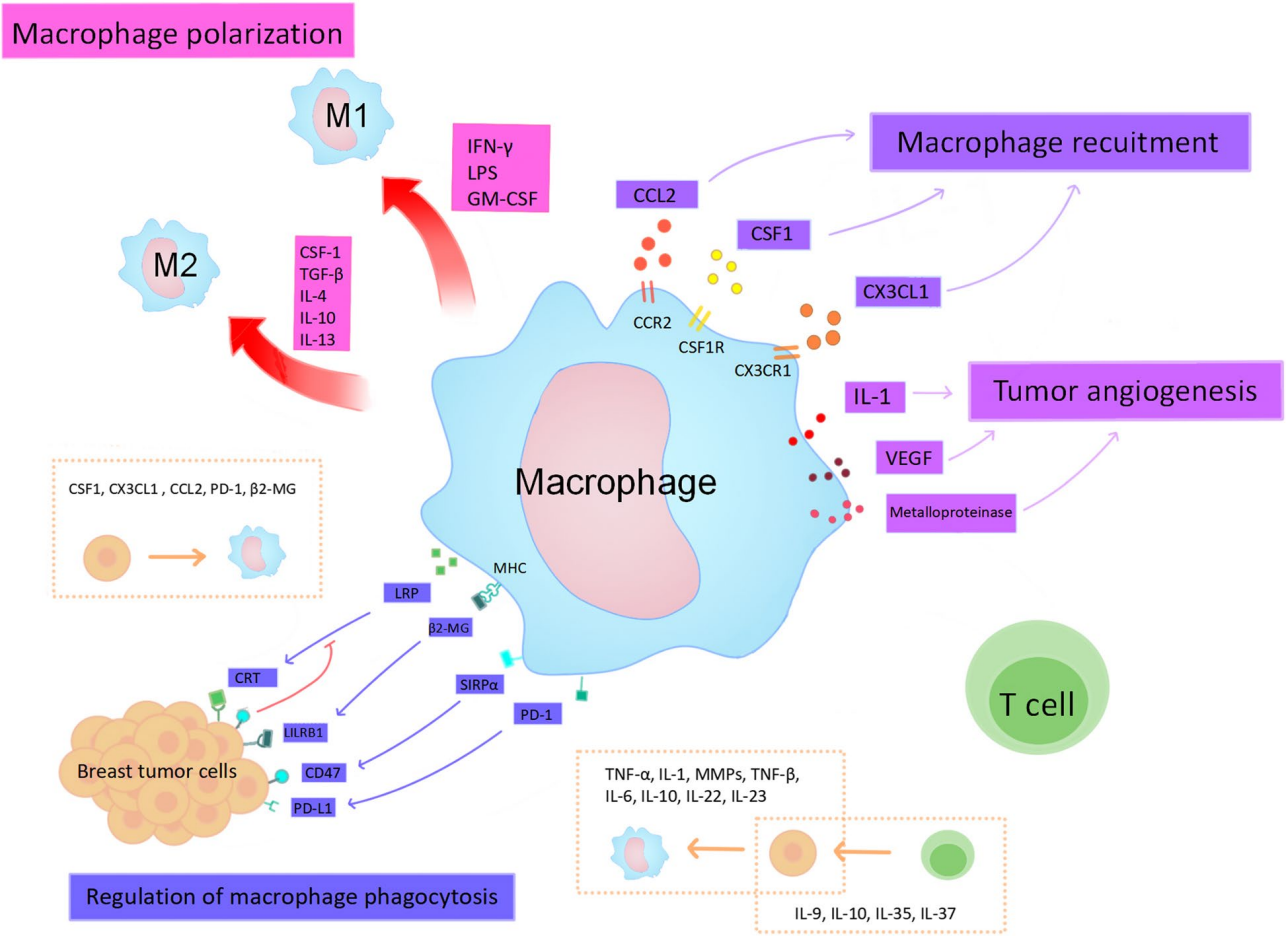
The breast tumor microenvironment regulates TAM in different aspects and the nteraction of TAM with breast tumor cells. There are four main areas. 1) Polarization, regulation of M1 polarization by IFN-γ, LPS, GM-CSF, and M2 polarization by CSF-1, TGF-β, IL-4, IL-10, IL-13; 2) recruitment, recruitment of TAM via CSF1, CX3CL1, CCL2; 3) tumor angiogenesis, VEGF, IL-1, Metalloproteinase to promote blood vessel growth. (4) Regulation of TAM phagocytosis by modulating CRT/LRP, LILRB1/β2-MG, CD47/SIRPα, PD-1 / PD-L1 affects TAM phagocytosis.The yellow dashed box depicts the interaction between TAM and breast cancer cells, and the effect of T cells on breast cancer tumor cells

In addition, TAM activation receptors affect TAM phagocytosis based on the regulation of immune checkpoints on the surfaces of tumor cells. The tumor cell surface expresses calreticulin, which interacts with TAM low-density lipoprotein receptor-associated protein (LRP) and emits pro-phagocytic signals. The inhibition of CD47 can block this process [79]. The expression of programmed cell death protein 1 (PD-1) suppresses multiple immune cells, including TAMs. Its binding to programmed cell death ligand 1 (PD-L1) on the surface of tumor cells renders cancer cells insensitive to the effects of tumor cell attack. PD-1/PD-L1 blockade is an effective way to restore the anti-tumor effect of TAM [83]. The β2-microglobulin (β2-MG) expressed by major histocompatibility complex (MHC) class I controls the phagocytosis of TAMs. It can interact with the LILRB1 inhibitory receptor on the surface of tumor cells to prevent being killed by phagocytosis [84]. The inhibition of the CD47-SIRPαsignaling pathway impairs the cytotoxicity of TAMs to tumor cells. In the MCF7 breast cancer cell line, CD47 increases gene transcription through NF-κB signaling, and blocking TNFαsignaling can inhibit CD47 expression. Drugs have been shown to inhibit SIRPαsignaling by blocking FcγR at the CD47 site using ADCC action [85]. Abundant cytokines in the tumor microenvironment can influence TAM growth and metabolism, mediating the formation of TAMs. On the other hand, TAMs can also act on the tumor microenvironment by regulating cytokines (Fig. 2).

### Regulation of cytokines and chemokines in the breast microenvironment by TAM

In addition to immune cells, there are various kinds of cells in the breast tumor microenvironment, including mesenchymal cells, fibroblasts, endothelial cells, and adipocytes. The first two together form the connective tissue. These cells, in interaction with tumor cells, secrete proteases and pro-tumor factors that affect the recruitment and metabolism of tumor cells [86]. TAMs alter the tumor microenvironment by secreting or modulating these cytokines, promoting increased blood flow, angiogenesis, and other inflammatory responses during cellular pyroptosis.

Tumor necrosis factor-α (TNF-α) is a cytokine co-secreted by TAMs and tumor cells. It plays an important pro-inflammatory role in the tumor microenvironment [87]. In fact, TNF-α increases the activation of the NF-κB signaling pathway in the endoplasmic reticulum and promotes estrogen synthesis. Meanwhile, TNF-α promotes prostaglandin (PGE2) synthesis and angiogenesis through the upregulation of synthase and promotes breast tumor growth through various pathways [88]. TAMs can secrete multiple matrix metalloproteinases (MMPs) and forms a complex network in the immune microenvironment. They can affect cancer development, embryonic development and wound healing [89]. MMPs have the ability to degrade extracellular matrix (ECM) proteins; remodel the tumor stroma; and promote the activation of cytokine production, such as that of IL-1β. This is related to the proliferation, invasion, and metastasis of tumor cells [90]. Transforming growth factor β(TGF-β) is associated with the invasion and metastasis of breast cancer [91]. TGF-β induces epithelial-mesenchymal transition (EMT), orchestrating a complex transcriptional network that stimulates the early growth and metastatic spread of tumor cells [92]. It has been shown that the novel plant extract rhodopsin can block the action of EMT induced with TAMs by inhibiting TGF-β signaling in breast cancer cells. In turn, this affects cancer stem cell (CSC) production, reduces TAM aggregation and polarization, and has an anti-tumor role [93]. The cytokine IL-1 has potent anti-inflammatory effects. IL-1 alpha is formed via cleavage by proteases (calpain II, caspase-1, and granzyme B). IL-1β requires transcription to be stimulated by the TNF or NF-κB signaling pathways and then activated by inflammatory vesicle complexes. These pathways can transmit downstream inflammatory signals, participate in inflammatory responses and immunosuppression, and provide a favorable environment for breast tumor cell growth [94]. TAMs-derived IL-6, IL-10, IL-22, and IL-23, together with T-lymphocyte-derived IL-9, IL-10, IL-37, and IL-35 and other cytokines, regulate the immune microenvironment of breast cancer tumors. Among these, IL-6 can maintain the activity of mammary stromal cells [95]. IL-10 increases the number of TAMs and T cells and promotes the expression of immunosuppressive proteins [96]. IL-22 stimulates breast cancer cell proliferation via epithelial proliferation and immunosuppressive effects [97]. IL-23 can activate transcriptional activator 3 (STAT3), which is involved in the growth and metastasis of many malignant tumors [98]. Studies on the prognosis of breast cancer tumors have shown that CHMP6, a pyroptosis-death-associated gene, is significantly downregulated in breast cancer. In contrast, the expression of IL-18, another pyroptosis-death-related factor, was significantly upregulated, which was not the same as the prognostic outcome in other cancers [24]. This could be due to a complex inflammatory microenvironment. Relevant cytokines in the tumor microenvironment are able to respond to various stimuli related to cell proliferation under the secretion or regulation of TAMs. These factors also promote breast cancer tumor growth by potentially affecting the pyroptosis mechanism through various pathways, which provides targets for in-depth studies of immunotherapy targeting TAMs.

### Chemotherapy drugs and breast cancer pyroptosis

With the increasing research on pyroptosis and malignancy, a variety of chemosynthetic drugs have been developed and applied to modulate the pyroptosis pathway to inhibit tumors. In the table, we summarize the chemotherapeutic agents that target breast cancer pyroptosis, as well as their targets and mechanisms (Table 1).

**Table 1:** List of chemotherapeutic agents targeting breast cancer pyroptosis

FINs	Targets	Mechanisms
xihuang pill (XHP) [99]	cAMP / PKA	Regulation of cAMP/PKA signaling pathway upregulates caspase-1,GSDMD
dihydroartemisinin (DHA) [100]	AIM2/ caspase-3/DFNA5	AIM2 activation causes cleavage of caspase-3, which in turn increases the expression of DFNA5
Quercetin [101]	PYD, ASC, NLRP3	Down-regulation of PYD, ASC, NLRP3, and inhibition of caspase-1 expression
Docosahexaenoic acid (DHA) [102]	caspase-1, gasdermin D	caspase-1 activates the cleavage of gasdermin D and increases the release of IL-1β
Benzenesulfonimide PPARα antagonists [103]	caspase-3, PPAR, Nrf2	Increases caspase-3 cleavage, affects the Nrf2/PPARα molecular axis, and increases superoxide anion production
nobiletin [104]	miR-200b/JAZF1\/NF-κB	Promoting miR-200b overexpression inhibits JAZF1 while promoting activation of NF-κB and GSDMD
andrographolide (ANDR) [105]	caspase-8	activation of caspase-8, FLIP (FLICE-like inhibitory protein) and XIAP (X-linked apoptosis inhibitory protein)
tubulysins [106]	caspase-3, GSDME	Antibody drug conjugate (ADC) therapy co-administered tubulysins to promote caspase-3 cleavage of GSDME
3-acyl isoquinolin-1(2H)-one [107]	GSDME	Induction of G2 phase block, apoptosis and GSDME-mediated pyroptosis in breast carcinoma
Triclabendazole [108]	caspase-3, GSDME	Activation of caspase-3 induces GSDME-dependent pyroptosis
RIG-I Agonists [109]	caspase-1, caspase-10	Activation of caspase-1 to cleave GSDMD
Spatholobus suberectus Dunn percolation extract (SSP) [110]	caspase-4, caspase-9, GSDME	Upregulation of inflammatory vesicles and ROS, activation of caspase-4 and caspase-9 cleavage of GSDME
Ganoderma lucidum extract (GLE) [111]	caspase-3, GSDME	Activates caspase-3/GSDME signaling pathway and induces immune response
Tetraarsenic hexoxide [112]	ROS, GSDME	Increased ROS production and GSDME-mediated pyroptosis

In clinical trials, xihuang pill (XHP) has been shown to induce pyroptosis of breast cancer cells by activating the cAMP/PKA signaling pathway, thereby inhibiting cancer cell proliferation, invasion, and migration [99]. A class of derivatives derived from the herb Artemisia annua, dihydroartemisinin (DHA), acts on processes such as cell proliferation, cell cycle arrest and angiogenesis, and also enables the pyroptosis-related factors caspase-3 and DFNA5, enhancing the sensitivity of breast cancer cells to the drug [100]. Quercetin, the active ingredient from the Protracted Anti-Aid Detoxification Formula, has antitumor activity in a variety of cancers and inhibits caspase-1-mediated scorching by downregulating PYD, ASC, and NLRP3 in breast cancer [101]. Docosahexaenoic acid (DHA) causes an increase in caspase-1 and GSDMD activity in breast cancer cells, triggering pyroptosis and inhibiting breast cancer growth [102]. Benzenesulfonimide PPARα antagonists block the link between the peroxisome proliferator-activated receptor (PPAR) and Nrf2 pathways in cancer, affecting the cell cycle at the G2/M checkpoint, reducing cancer cell proliferation, and increasing caspase-3 cleavage to promote pyroptosis [103]. Nobiletin, a polymethoxylated flavonoid found in citrus fruits, regulates the miR-200b/JAZF1 axis through the NF-κB signaling pathway to cause breast cancer cell pyroptosis and inhibits cell proliferation in a dose-dependent manner [104]. Andrographolide (ANDR) has been shown to be effective in the treatment of ovarian and breast cancers, inducing activation of caspase-8,FLIP and XIAP, promoting tumor pyroptosis and apoptosis, and inhibiting invasive metastasis [105]. Tubulysins can inhibit mitosis while inducing GSDME-mediated pyroptosis by promoting caspase-3. In clinical settings Tubulysins are used in combination with Antibody drug conjugate (ADC) therapy for the treatment of breast cancer, targeting breast cancer cells and mitigating drug toxicity [106]. 3-acyl isoquinolin-1(2H)-one is an effective antitumor agent that can treat breast cancer through multiple pathways. It can promote LDH release and induce GSDME cleavage to trigger pyroptosis, induce G2 phase blockade to inhibit cancer cell proliferation, and promote apoptosis through the mitochondria-induced intrinsic apoptotic pathway [107]. Triclabendazole, an imidazole anthelmintic that was later found to be structurally similar to chemotherapeutic drugs, has better anticancer activity and can activate caspase-3 to induce GSDME-dependent pyroptosis [108]. RIG-I agonists can activate the intrinsic immune effector RIG-I in breast cancer cells, increase the immunogenicity of the tumor, activate caspase-1 cleavage of GSDMD in vitro, and are a viable approach for the treatment of breast cancer [109]. Spatholobus suberectus Dunn percolation extract (SSP), the active ingredient extracted from chickweed, has a tumor-killing effect, which is mainly caused by the upregulation of inflammatory vesicles and ROS, activation of caspase-4 and caspase-9 to cleave GSDME causing pyroptosis and rupture of cell membranes [110]. Ganoderma lucidum extract (GLE) has immunomodulatory, anti-inflammatory and anti-angiogenic effects in cancer therapy, and studies have shown that GLE in breast cancer activates caspase-3 to cleave the GSDME protein and release inflammatory factors to cause an immune response [111]. Tetraarsenic hexoxide is a chemotherapeutic agent for clinically advanced cancer patients that increases ROS production and GSDME-mediated pyroptosis and inhibits invasive metastasis of breast cancer cells [112].

Most chemotherapeutic agents can affect the immune microenvironment, inducing tumor cells to produce macrophage recruitment factors such as CSF1, which enhances macrophage infiltration (37). TAM are capable of influencing tumor vasculature and lymphatic vessel formation and can regulate the immune microenvironment and influence the pyroptosis pathway by producing a large number of immune factors and chemokines. Therefore, combining targeted TAM therapy with chemotherapeutic agents provides a viable direction for breast cancer treatment.

### TAM-targeted therapy

TAMs play a key role in the development of breast tumors and are an effective target for the control and treatment of breast cancer. By blocking tumor-associated cytokines and reducing angiogenesis through TAM-targeted therapy, there is hope of inhibiting cell scorching and thus slowing down breast tumor progression. Treatment strategies for TAMs fall into two categories. The first is the inhibition of tumor-promoting macrophages (M2-type macrophages), mainly by inhibiting their recruitment and proliferation. The second is the activation of antitumor macrophages, the induction of TAM polarization toward M1-type macrophages, or the reprogramming of M2-type macrophages into M1 cells [113]. The presence of multiple chemokines in the tumor microenvironment affects TAM recruitment. CCL2 is an M2 aggregation chemokine closely related to breast cancer, and anti-CCL2 treatment reduces the amount of M2 in tumors and limits the metastatic spread of cancer cells. However, extra caution should be taken when considering anti-CCL2 therapies because overtreatment may lead to vascular hyperplasia and poor prognosis [114]. The disruption of the CX3CL1/CX3CR1 axis is also an effective strategy with which to inhibit M2 aggregation, providing a direction for TAM-targeted therapy [115]. The administration of PLX3397 (pexidanib), an antagonist of CSF-1R, caused the depletion of TAMs and delayed tumor regeneration after radiotherapy [116]. Although the pro-tumor function of TAMs often brings about adverse results, how to make good use of the positive effect of TAMs against tumors is a question worth exploring. Multiple signaling molecules that regulate TAM polarization can reverse the TAM phenotype, which makes TAM reprogramming possible. The inhibition of transcription factor STAT3 expression can reprogram TAM to mediate anti-tumor immunity. STAT3 inhibitors are still in development. Studies have shown that CD163-targeted drugs can inhibit STAT3 activation at the protein level [117]. The use of anti-CD47 antibody enhances phagocytosis and directs TAMs from the M2 phenotype to the M1 phenotype [118]. TAM-targeted nanoparticles are capable of reprogramming TAMs for degradation and modulation using relevant ligand-modified nanomaterials, opening up a new pathway for cancer therapy [119].It has been shown that several components of myrrh extract showed moderate cytotoxicity against breast cancer cell lines, as compared to rectal cancer cell lines, while being able to increase M1 phenotypic markers. Therefore, multi-targeted drug therapy can better utilize such biological properties for immunomodulatory effects [95]. Drug resistance treatment is limited by the tumor microenvironment, so targeted TAM therapy also has a better effect on breast cancer drug resistance mitigation, and there is hope for in-depth study [120].

### Summary

In summary, there is a complex interaction between TAMs and tumor cells, which indirectly affects the pyroptosis process via the secretion of cytokines. In recent years, an increasing number of studies have shown that pyroptosis is an important regulator of the tumor microenvironment.

An analysis of 33 focal death-related genes in breast cancer tissues and the determination of their expression levels revealed that five were expressed at higher levels than in the surrounding tissues. This provides direction for finding breast cancer biomarkers [121]. A comparative analysis of tumor cell focal death patterns and TME cell infiltration characteristics through genomic studies has led to the conclusion that the two are inextricably linked. Tumor-associated macrophages are an important component of the TME and also belong to the second line of defense of the innate immune system. These TAMs are also essential in cellular and humoral immunity, secreting a variety of cytokines and playing a wide range of regulatory roles [122]. The complex cytokine networks mediating different signaling pathways also provide targets for tumor-targeted therapies. Relieving immunosuppression, blocking the binding of corresponding cytokines to receptors, and regulating the immune microenvironment in the anti-tumor direction are very important parts of inhibiting tumor development. Antitumor vectors and vaccines targeting immune cells have been reported [123]. It takes full advantage of the effectiveness of the immune system. Therefore, an in-depth study of the mechanism of interaction between TAMs and the immune microenvironment is necessary.

## Data Availability

The study did not produce any new or raw data.
